# Protein content prediction in single wheat kernels using hyperspectral imaging

**DOI:** 10.1016/j.foodchem.2017.07.048

**Published:** 2018-02-01

**Authors:** Nicola Caporaso, Martin B. Whitworth, Ian D. Fisk

**Affiliations:** a Campden BRI, Chipping Campden, Gloucestershire GL55 6LD, UK; b Division of Food Sciences, University of Nottingham, Sutton Bonington Campus, LE12 5RD, UK

**Keywords:** Near-infrared spectroscopy, Wheat protein, Cereals, Rapid measurement, Chemical imaging, Single kernel assessment, Hyperspectral imaging

## Abstract

Hyperspectral imaging (HSI) combines Near-infrared (NIR) spectroscopy and digital imaging to give information about the chemical properties of objects and their spatial distribution. Protein content is one of the most important quality factors in wheat. It is known to vary widely depending on the cultivar, agronomic and climatic conditions. However, little information is known about single kernel protein variation within batches. The aim of the present work was to measure the distribution of protein content in whole wheat kernels on a single kernel basis, and to apply HSI to predict this distribution.

Wheat samples from 2013 and 2014 harvests were sourced from UK millers and wheat breeders, and individual kernels were analysed by HSI and by the Dumas combustion method for total protein content. HSI was applied in the spectral region 980–2500 nm in reflectance mode using the push-broom approach. Single kernel spectra were used to develop partial least squares (PLS) regression models for protein prediction of intact single grains.

The protein content ranged from 6.2 to 19.8% (“as-is” basis), with significantly higher values for hard wheats. The performance of the calibration model was evaluated using the coefficient of determination (R^2^) and the root mean square error (RMSE) from 3250 samples used for calibration and 868 used for external validation. The calibration performance for single kernel protein content was R^2^ of 0.82 and 0.79, and RMSE of 0.86 and 0.94% for the calibration and validation dataset, enabling quantification of the protein distribution between kernels and even visualisation within the same kernel. The performance of the single kernel measurement was poorer than that typically obtained for bulk samples, but is acceptable for some specific applications. The use of separate calibrations built by separating hard and soft wheat, or on kernels placed on similar orientation did not greatly improve the prediction ability. We simulated the use of the lower cost InGaAs detector (1000–1700 nm), and reported that the use of proposed HgCdTe detectors over a restricted spectral range gave a lower prediction error (RMSEC = 0.86% vs 1.06%, for HgCdTe and InGaAs, respectively), and increased R^2^ value (R_c_^2^ = 0.82 vs 0.73).

## Highlights

• HSI was applied for non-destructive prediction of total protein content in wheat kernels.
• Above 2100 wheat kernels were taken from ~200 batches and individually analysed.
• PLS regression models had R^2^ = 0.82 and prediction error lower than 0.93%.
• Protein distribution had wide range (6–20%) and was visualised by applying the calibration.
• The performance of HgGcTe was superior to the one built by simulating InGaAs sensors.

## 1 Introduction

Wheat is a staple commodity worldwide, used both for human consumption and for feed. Among wheat quality parameters, physical condition, moisture content, kernel hardness, Hagberg Falling Number (an indirect measurement of the effect of α-amylase activity in flour and ground wheat), and protein content are the most important. Protein content is important because it influences the technological performance in baked products, especially gluten formation for bread production, in combination with protein quality which is determined by varietal choice. Protein content has a significant impact on the final price, and many countries adopt it as a critical criterion to define wheat price.

Near-infrared (NIR) spectroscopy is a non-destructive and rapid method that can be used to investigate the chemical properties of complex food matrices and intact seeds or grains (Fox & Manley, 2014). The approach is based on the interaction of light radiation with the sample, in particular on molecular overtone and combination vibrations. NIR spectroscopy strongly relies on chemometrics for prediction of properties or classification of samples based on multivariate regression models, typically combined with spectral pre-treatment techniques. Common spectral pre-treatments aim to remove some interference due to the physical properties of the analyte, for example the particle size. These methods include multiplicative scatter correction (MSC), standard normal variate (SNV) and de-trending. A second category of spectral pre-treatments is based on the application of derivatives, including a smoothing step often using the Savitzky-Golay convolution method (Geladi, 2003, Rinnan et al., 2009).

NIR spectroscopy measurement of protein content in wheat was initially based on batches of ground wheat or flours (Osborne, 1984). The feasibility of NIR prediction in batches of whole kernels without the need of grinding has been also demonstrated (Williams & Norris, 1987). Whole-grain applications became attractive to breeding programmes and industry due to the non-destructive scanning of samples and the speed of analysis, with the possibility of on-line or in-line data acquisition (Osborne, 1987, Williams and Sobering, 1993). Total protein prediction models based on NIR and visible spectroscopy of batches of whole wheat have high correlation coefficients (Cozzolino, Delucchi, Kholi, & Vázquez, 2006). However, the traditional NIR spectroscopy approach is based on bulk grains, and thus no indication of the protein variability among kernels is given (Bramble, Dowell, & Herrman, 2006). Some authors have tested single kernel NIR analysis, and applied it successfully to measure wheat protein content in transmittance mode (Delwiche, 1995). More recently, reflectance NIR spectroscopy was also applied, due to its better applicability at industrial level, and also tested for single kernels (Bramble et al., 2006).

The potential of single kernel NIR analysis strongly depends on its application and the quality parameter studied. For example it has limited potential to identify wheat varieties in breeding programmes, while more successful applications have been reported for physical grain quality determination, moisture, protein and kernel hardness measurement and loss of viability (Fox & Manley, 2014).

Protein variability among wheat kernels was reported for some USA wheat classes using NIR reflectance spectroscopy on a single kernel basis (Delwiche, 1998), and it was demonstrated that protein prediction of the batch can be improved by averaging a few hundred kernels from single kernel measurement (Delwiche & Hruschka, 2000). A single kernel characterisation of some European wheats was also performed by NIR transmittance spectroscopy (Nielsen, Pedersen, & Munck, 2003).

The possibility of high speed classification of single kernels for quality attributes is relevant in cereal breeding programmes, to improve the product quality. Whilst single kernel protein calibrations have been demonstrated, presentation of kernels individually results in practical difficulties for rapid analysis of bulk samples. Hyperspectral imaging (HSI) provides a potential approach to enable single kernel data to be acquired for larger numbers of kernels.

HSI combines NIR spectroscopy and digital imaging to give information about the spatial distribution of compounds. HSI creates three-dimensional “hypercube” datasets composed of two spatial dimensions and a single spectral dimension representing NIR spectra for each pixel of the image. As for bulk NIR spectroscopy, HSI heavily relies on chemometrics to extract useful chemical information from the hypercube (Gowen, O'Donnell, Cullen, Downey, & Frias, 2007). It has been applied successfully to measure the distribution of chemical composition in a wide range of food, including meat, fish, fruits, vegetables, and several applications to cereals (Gowen et al., 2007, Wu and Sun, 2013). These include exploratory tests of HSI to measure or predict the milling quality of soft wheat (Delwiche, Souza, & Kim, 2013).

Although NIR calibrations show good performance for measurement of protein content in bulk wheat samples and are commonly applied at industrial level for laboratory and online measurement, limited work has been done on the application of HSI for wheat protein analysis. HSI offers potential advantages for assessment of uniformity in wheat and other granular food materials, united with the advantage of NIR spectrometry being contactless and rapid.

Therefore, the aim of our study was to develop an HSI calibration for total protein content in whole wheat kernels on a single grain basis and to assess the typical uniformity present in commercial wheat samples, and thus to apply the calibration to visualise the protein distribution within single kernels.

## 2 Material and methods

### 2.1 Wheat samples

Samples were obtained from a wide range of suppliers, mainly millers and breeders from the UK. Examples of Canadian, French, Italian, German and Eastern European wheat samples were also included. Samples came from the 2013 and 2014 harvests. They were selected to obtain a wide variation in terms of cultivars, environment and agronomic conditions, also including some genotypes from breeding trials and not yet registered or under registration. A total of approximately 190 wheat samples were used for the present experiment. From each sample, 10–12 kernels were randomly selected to be used for the analyses. Each kernel was presented for HSI measurement in both crease-up and crease-down orientations, resulting in a total of ∼4200 kernel spectra. Each kernel was then analysed by the Dumas method to determine its protein content. The spectra and reference protein values were used for development and validation of the calibration.

### 2.2 Hyperspectral imaging

Data was acquired using a laboratory-scale hyperspectral imaging system described by Millar, Whitworth, Chau, and Gilchrist (2008) and Caporaso, Whitworth, and Fisk (2016). The instrument was supplied by Gilden Photonics Ltd. (Glasgow, U.K.) and includes a SWIR spectral camera (Specim Ltd., Oulu, Finland) containing a cooled 14 bit 320 × 256 pixel HgCdTe detector and N25E spectrograph providing 256 spectral bands over a wavelength range of ∼980–2500 nm with a spectral resolution of about 6 nm. Samples were presented on a moveable sample stage and imaged using a push-broom approach. The camera was mounted above the stage at a distance of 220 mm and a 31 mm focal length lens was used, resulting in a swathe of 35 mm and a pixel size of 0.109 mm for 320 spatial pixels. Images were acquired at a rate of 100 frames s^−1^, using a stage translation speed of 10.9 mm s^−1^, providing the same spatial resolution parallel and perpendicular to the scan direction. A single 500 W incandescent illumination source was used for the first ∼1000 kernels, and 2 lamps were used for the remaining samples. SpectralCube 3.0041 software (Specim) was used to control the camera and translation stage. The camera shutter was automatically closed for 1 s at the end of each scan and ∼100 frames were recorded to establish the baseline signal of the detector (black reference). Separate scans of approximately 100 frames were also recorded for a white PTFE reference material with approximately 100% reflectance across the entire measured spectral range (white reference).

Wheat samples were presented with grains arranged in two rows on a black, NIR-absorbent plastic tray, and the hypercubes were obtained in diffuse reflectance mode. Images were first acquired for the dorsal side of the kernels, and the kernels were then manually rotated and a second image acquired for the ventral side. To minimise heating of the sample, the lamps were only turned on for the duration of the scan.

### 2.3 Analysis of hyperspectral data

Hyperspectral images were analysed using ENVI 5.2/IDL 8.4.1 software (Harris Geospatial Solutions). The following operations were carried out to obtain NIR spectra for each kernel from the hypercubes:

1. Calculation of reflectance data from the raw image by subtraction of the black reference and normalisation by the white reference. Absorbance values were then calculated as log_10_(1/reflectance);
2. Removal of spikes from absorbance data. These are caused by factors such as bad pixels in the detector. They were identified as single pixel outliers by comparing with the median absorbance of neighbouring pixels;
3. Segmentation of kernel images from the background tray using a binary threshold criterion of absorbance at 1186 nm < 0.9;
4. Identification of kernels and indexing according to position on the tray;
5. Calculation of the mean absorbance spectrum for each kernel, and export for statistical analysis.

Once the calibration equation had been created, it could be applied for rapid prediction of the protein content of test samples, using a further ENVI-IDL program. Hyperspectral images of these samples were acquired in the same manner, but with no restriction on the orientation, position or number of kernels. Absorbance values were calculated for each pixel as above and kernel images were segmented from the pixels belonging to the tray. The program applied the chosen spectral pre-treatments and applied the calibration coefficients to calculate a protein value for each pixel in the kernels. This enabled the spatial variation of predicted protein content to be visualised within a single grain or average protein values to be calculated for each kernel or sample. For some types of pretreatment, different single kernel values may be obtained depending on whether the calibration is applied before or after calculation of average values for the pixels in the kernel (Caporaso et al., 2016).

### 2.4 Reference analysis for protein content (Dumas combustion method)

Dumas combustion was used to determine the protein content of each wheat kernel. As part of this determination, the mass of each kernel was also measured. Dumas protein values were measured on an as-is moisture basis (N x 5.7) according to ISO/TS 16634-2 (2009). A Leco FP-628 (LECO, Stockport, UK) instrument was used to perform the analysis. To minimise moisture changes between HSI and Dumas measurements, the samples were analysed on the same day and exposure to high temperature was avoided. Two samples of flour with low (∼10.2%) and high (∼12.4%) protein values were systematically used to check for possible drift during the analysis. The final results were expressed as total protein content on an “as-is” basis. The repeatability of the method was below 0.1%, while the reproducibility (method standard deviation) was 0.6% for low protein sample and 0.7% for high protein check sample.

### 2.5 Data analysis and PLS calibration

The single kernel NIR reflectance spectra and corresponding Dumas protein measurements were processed using The Unscrambler X 10.3 (Camo, Norway) to develop calibrations for protein content based on PLS regression analysis. Approximately 2100 individual wheat kernels were scanned both dorsally and ventrally, resulting in a dataset of ∼4200 mean spectra. From these, spectra for approximately 80% of randomly selected grains were used as the calibration set, while the remaining 20% constituted the independent validation set. Spectral outliers were detected by considering the residual versus leverage plots and residual variance once the PLS model was developed, while some outliers from reference measurements were removed in case of error during the Dumas measurement. The optimal number of latent variables for the model was determined by using the cross-validation dataset, and leaving the software to choose the best latent variable, or principal component. The model was then recalculated using the selected latent variable and applying the external validation set.

The following spectral pre-treatments and combinations of these were tested to assess their effect on the calibration performance. Scatter correction pre-treatments including standard normal variate (SNV), de-trending and multiplicative scatter correction (MSC) were used to minimise the non-linear effect of light scatter due to particle size differences among samples (Barnes, Dhanoa, & Lister, 1989). Different orders of derivative were also tested, mainly applying first and second derivatives using 5-point Savitzky-Golay smoothing. The coefficient of determination (R^2^), the slope, bias, ratio of performance deviation (RPD) and root mean square error of calibration (RMSEC) and prediction (RMSEP) were used to describe the fitness of models generated.

## 3 Results and discussion

### 3.1 Single kernel protein variability in UK wheats

**Fig. 1:**
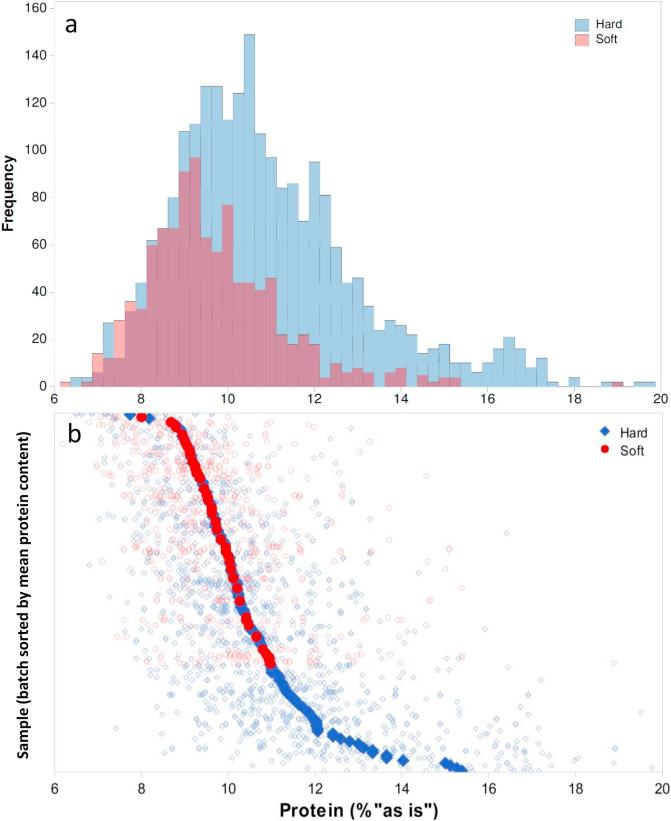
**a**) Distribution of total protein content in single wheat kernels, on the full dataset used in the present experiments, by separately showing the hard and soft wheats. **b**) Dispersion of protein content at single kernel basis (empty symbols) and average of the batches (filled symbols).

Table 1 reports the descriptive statistics of the samples used in the present study. The statistics for the validation and calibration datasets are shown separately, reporting the protein content and single kernel weight. Fig. 1 shows the distribution of single kernel protein content measured by the Dumas method for the UK samples used in this study. Fig. 1b describes the distribution of reference protein content in single kernels by also displaying the kernels belonging to the same batch, and the average protein content for each batch. In this way, the total protein content is shown, alongside the within-batch variability. The minimum protein value was slightly above 6.1%, while the maximum was 19.8%. From the protein distribution plot, 42% of kernels had a protein content in the range 8–10%, which is generally considered as medium-low protein content for wheat batches, while in our case it is shown at single kernel level. Moreover, in certain countries such as the UK, a wheat batch cannot be classified in the superior category when its batch average protein content is below a certain value, e.g. 13% in the case of Group 1 wheats according to the nabim scheme (http://www.nabim.org.uk/ last access: 15.02.2017). However, the main focus for this paper was to understand the variability at single kernel level. As our samples also included some batches of Durum wheat and several batches of Canadian hard red wheat, grown especially for their high protein content, this is likely to be the reason for the non-Gaussian distribution observed for the whole dataset, with a few kernels showing a protein content above 16%, while the mean value of the batches did not reach 16% protein. This irregular distribution was expected, as several samples of hard wheat used as improver were analysed, e.g. Canadian or other hard wheats with high protein content, which in general are used in addition to UK wheat with moderate protein content.

**Table 1:** Statistical analysis for the parameters used in the present paper, separately showing the **a**) calibration and **b**) validation datasets. SD = standard deviation. CV = coefficient of variation.

	Mean	Range	Min	Max	SD	CV (%)
*a) Calibration dataset*						
Protein content (%)	10.59	13.62	6.15	19.77	2.07	19.55
Kernel weight (mg)	49.6	72.2	15.5	87.7	12.1	24.47
*b) Validation dataset*						
Protein content (%)	10.45	12.66	6.6	19.26	2.06	19.67
Kernel weight (mg)	49.5	65.5	17.4	82.9	12.0	24.30

The average protein content of the whole dataset was 10.56%, with a standard deviation of 2.07%, and a range from 6.15 to 19.77%. When discriminating according to the hardness class, hard wheats had higher average protein content of 10.5%, compared to 9.5% in soft wheat. This difference was expected as hard wheat cultivars include those produced for bread-making for which higher protein content is desirable. On the contrary, soft wheat cultivars generally present lower protein content and include those favoured for biscuit production (Payne, 1987).

In addition to genetic factors, one of the most relevant environmental factors affecting protein content in cereals is the nitrogen treatment, in terms of amount and timing of nitrogen fertilisation (Farrer, Weisz, Heiniger, Murphy, & White, 2006). The minimization of protein variability in wheats can be an important aspect in wheat research, for growers/farmers and for its importance in breeding programmes to obtain consistent quality traits.

Much information is available on the average protein content of bulk wheat samples, and this is widely used as an important specification for commercial trade and processing of wheat. However, limited information has been published on the natural variation in single kernel properties within samples, whereas this parameter is of potential interest for processors and breeders, e.g. to understand the effects of genetic traits and agronomic factors. This information could be applied to improve wheat quality in terms of crop homogeneity, to be used as an indicator of plant nutrition requirements with a consequent potential increase in productivity and protein content.

Some of the research published so far on wheat quality variability concerns samples in field trials using controlled soil characteristics, soaking rate and agronomic practices. We instead sampled a wide range of wheat cultivars including trial samples and commercial samples from several locations, grown under common agronomic practices. This provides an overview of the expected variability under production conditions, which is representative of the wheat industry, especially for the UK.

The variability of protein content on a single wheat kernel basis has been reported by Delwiche (1998) for US wheats. Depending on the wheat class, the average protein content ranged from circa 8.8% (w/w “as-is”) for hard white wheat up to 13.2% for hard red spring wheat, with standard deviations in the range 1.6–3.0%. A wide range of protein content within a single batch has been also reported for other cereals, e.g. Fox, Kelly, Sweeney, and Hocroft (2011) studied the protein variability in single barley kernels by NIR spectroscopy and reported a range from 7.3 to 16.6% (as-is).

In our case, the standard deviation (SD) observed in the whole dataset was 2.1% (as-is). Generally, a greater SD was observed for hard wheats (2.2%), than soft wheats (1.6%). The average kernel weight was 49.6 mg, with a standard deviation of 12.0 mg, and we also investigated the relationship between the kernel weight and protein content. The existence of a correlation between wheat kernel size or weight and protein content has been under debate, as it depends on the source, in fact it might vary depending on the focus at a single sample, among commercial samples or as a result of agronomic variables. Bramble et al. (2006) and Wilkins, Douglas, and Churchill (1993) reported a moderate negative correlation between these two factors considering 500 wheat kernels, while Delwiche, 1995, Delwiche, 1998 and Dowell et al. (1997) did not observe any correlation. In our case, the Pearson correlation value for the kernel weight and total protein content (% “as-is”) was 0.018, for >2100 kernels analysed, with a strong statistical significance (p < 0.001), therefore in our dataset the correlation is significant but it is extremely weak. Delwiche (1998) reported the absence of any relationship between single-kernel protein content and the weight of kernels. Therefore, whilst a slightly better correlation might be observed for samples under a limited range of conditions, in bigger datasets including several variables such as hard and soft wheats, several harvesting years and geographical origin, a clear correlation is not apparent.

### 3.2 Protein prediction by HSI and application of the calibration

Table 2a reports the performances of the PLS models built from the spectra (spectral region: 1060–2500 nm) of >2000 individual wheat kernels, using several spectral pre-treatments. As each kernel was scanned on the dorsal and ventral side, two average spectra were acquired for each sample, thus giving more than 4000 spectra.

**Table 2:** Performance summary of hyperspectral imaging PLS regression models based on near-infrared reflectance spectroscopy data for **a**) protein content prediction in single wheat kernels and **b**) single kernel weight prediction.

*a) Total protein*										
Pre-processing	Calibration (n = 3250)				Validation (n = 868)					
	Slope	Bias	RMSEC	R^2^	Slope	Bias	RMSEP	R^2^	PC	RPD
Raw data	0.786	−0.004	0.949	0.786	0.799	−0.030	1.005	0.762	18	2.06
Normalisation	0.769	0.000	0.984	0.769	0.779	−0.050	0.990	0.768	16	2.09
MSC	0.810	0.000	0.897	0.810	0.800	−0.067	0.968	0.779	15	2.14
SNV	0.800	0.000	0.920	0.800	0.791	−0.066	0.973	0.777	15	2.13
SNV + 1st derivative	**0.824**	**0.000**	**0.857**	**0.824**	**0.819**	**−0.030**	**0.944**	**0.790**	**12**	**2.19**
SNV + de-trend	0.802	0.000	0.915	0.802	0.792	−0.072	0.983	0.772	13	2.11
2nd derivative	0.750	−0.007	1.025	0.750	0.777	0.012	1.075	0.728	10	1.93


*b) Kernel weight*										
Pre-processing	Calibration (n = 3115)				Validation (n = 998)					

Slope	Bias	RMSEC	R^2^	Slope	Bias	RMSEP	R^2^	PC	RPD

Raw data	0.667	0.000	6.992	0.667	0.615	0.109	7.595	0.607	12	1.58
Baseline + de-trend	**0.682**	**0.079**	**6.817**	**0.682**	**0.650**	**−0.053**	**7.439**	**0.623**	**13**	**1.61**
MSC	0.618	0.004	7.510	0.618	0.567	0.052	7.976	0.567	10	1.50
SNV	0.656	−0.025	7.110	0.656	0.603	0.012	7.701	0.596	13	1.56
SNV + 1st derivative	0.645	0.000	7.208	0.645	0.610	−0.139	7.689	0.597	11	1.56
2nd derivative	0.663	0.000	7.023	0.663	0.634	−0.090	7.722	0.594	12	1.55

Spectral range applied: ∼1060–2494 nm. Abbreviations: RMSEC: Root mean square error of calibration; RMSEP: root mean square error of prediction; PC: principal component, or latent variable; MSC: multiplicative scatter correction; SNV: standard normal variate. RPD: ratio of performance deviation. Both first and second derivatives were calculated by also applying the Savitzky-Golay smoothing. The models shown in bold indicate the best performance. RMSEC and RMSEP are expressed in % for **a**, and in mg for **b** (“as is” basis).

**Fig. 2:**
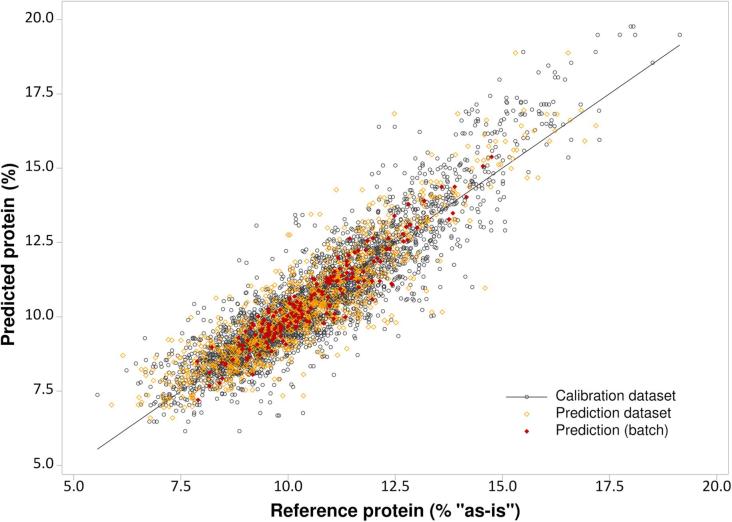
PLS regression model for total protein content in single wheat kernels (n = 3150 for calibration set and n = 1000 for validation set), by using the NIR spectral region ∼1060–2500 nm (pre-treatments used: SNV and first derivative calculated using Savitzky-Golay smoothing). Black: calibration dataset; yellow: external validation dataset (prediction); red: average batch values. For the colour identification please refer to the online colour version. R_c_^2^ = 0.916; R_cv_^2^ = 0.856; RMSEC = 0.876%; RMSECV = 0.507% for the batch; the other calibration performances are reported in Table 2.

From the average reflectance spectra obtained, it was possible to build PLS models for single kernel protein content with a root mean square error (RMSE) lower than 1%, both for the calibration and validation datasets, and a R^2^ of 0.79 and 0.76, respectively. The best performance was achieved for standard normal variate (SNV) treatment followed by first derivative calculation, resulting in R^2^ of 0.82 and 0.79 and a RMSE of 0.86 and 0.94%, for the calibration and validation datasets respectively. The result for this model is shown in Fig. 2. This performance for single kernels compares slightly better with typical results for bulk samples when the model is calculated for the 10–12 grains in a batch (filled symbols in the figure), by averaging the spectra and the reference protein value. In this case, the model shown gives R^2^ = 0.92 for the calibration dataset, with RMSEC = 0.39% and RMSECV = 0.51%.

**Fig. 3:**
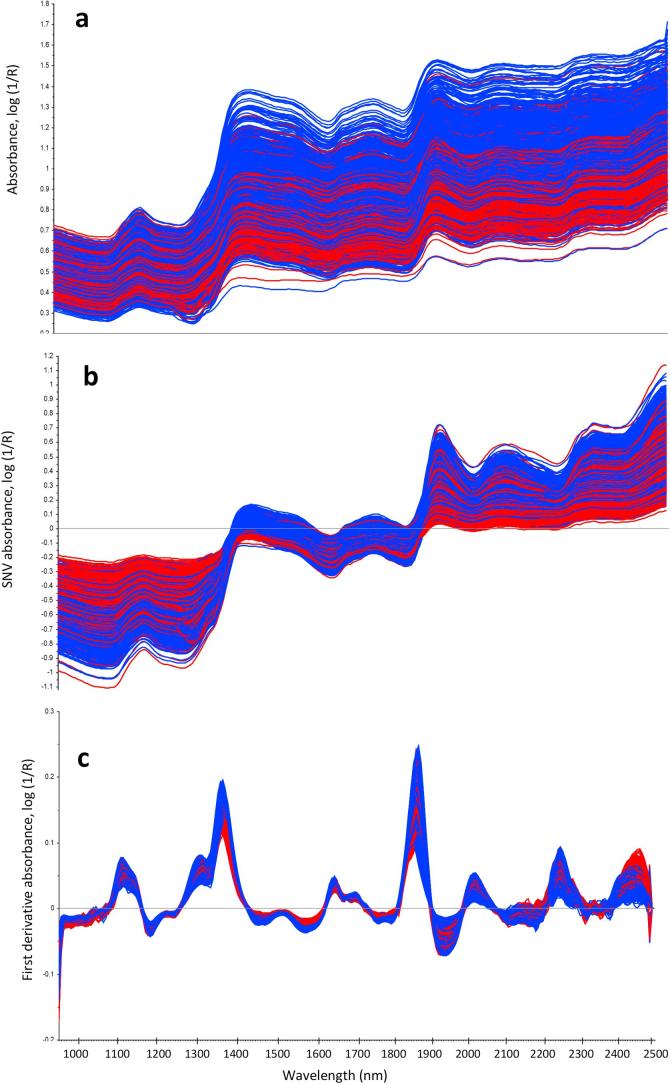
Mean NIR spectra for all wheat kernels: **a**) absorbance spectra; **b**) SNV-corrected spectra; **c**) spectra after SNV + first derivative calculation. Red: dorsal kernel side; blue: ventral side. For the colour identification please refer to the online colour version.

The models reported in Table 2 were built using an external validation dataset and not a cross-validation, which leads to the risk of overfitting calibration models (Martens & Dardenne, 1998). Some authors have overcome this issue by using a leave one batch out cross-validation (Schulmerich et al., 2012), but an independent validation offers more confidence in the prediction ability. From the obtained hypercubes, spectral pre-treatments are required to reduce scattering effects and the influence of the light source and sample presentation (Fig. 3). In our case the SNV + 1st derivative treatment resulted in the best model performance compared to the other treatments tested. Previous work investigated the effectiveness of spectral pre-treatment for NIR and HSI. For example, Agelet, Armstrong, Clariana, and Hurburgh (2012) discussed that, whereas SNV has been shown to minimise scattering effects, the combination and interaction of several factors producing scattering cannot be successfully addressed by SNV, as their calibration quality did not improve with SNV pre-treatment for single seed NIR measurements on soybean. However, in our case SNV appeared to be one of the most effective spectral pre-treatments, which can be attributed to differences in the shape and surface properties of the wheat kernels regarding the light scattering during the analysis. A generalisation on the spectral pre-treatment cannot be made as usually each study tested several pre-treatments and compared the performance of the models obtained, which strictly depends on the instrumentation used and sample presentation.

The calibration and prediction errors obtained in the present study are in line with previous literature on whole wheat kernels analysed by NIR instrumentation, and even with some papers dealing with protein in single wheat kernels. For example, Delwiche (1998) reported an SEC value of 0.494% for PLS calibration models based on reflectance NIR spectroscopy of different wheat classes, Cozzolino et al. (2006) reported SECV = 0.54% for bulk whole wheat and Bramble et al. (2006) reported errors of 0.32–0.51% for four wheat varieties with weight correction and 0.93 to 1.52% without.

The protein prediction models were previously reported to show differences in both the SECV and R^2^ values depending on the wheat cultivar tested. For instance, Bramble et al. (2006) reported a comparison of single-kernel prediction by reflectance NIR spectroscopy in four cultivars of hard red winter wheat, for which the R^2^ ranged between 0.79 and 0.93 with weight correction, and SECV values of 0.93–1.52% (protein range: 7.2–13.8%). Therefore, despite the fact that the authors did not apply HSI but just NIR, and they just used one wheat class, our model compared favourably. However, in our study, adjustment for kernel weight gave no improvement in the model. Using NIR in transmittance mode in the narrower spectral region 850–1050 nm, Delwiche (1995) reported a value of the coefficient of determination for protein prediction in wheat of 0.89, and a SEP of 0.45 (with a range of 0.4–0.9%), by analysing 94 samples of North American wheats.

The general lower prediction ability for single kernel HSI with respect to the conventional spectroscopy methods was previously highlighted by Delwiche et al. (2013), for three quality parameters of wheat, i.e. flour yield, softness equivalent and sucrose solvent retention capacity. For example, the authors reported that the coefficient of determination for softness equivalent was approximately 0.5 with HSI, and 0.6 for traditional NIR instruments. This may be due to additional random error coming from the single kernel determination, and for single kernel reference measurement, and potentially due to differences in detector performance. However, a calibration equation should be evaluated not just on the basis of equation performance itself, but also taking into consideration the advantages that NIR spectroscopy and particularly HSI brings over other analytical methods and even compared to bulk NIR spectrometers. Beside the rapid and non-destructive analysis, it gives the possibility to visualise the composition of a food product on a single grain basis up to a single pixel distribution (Fox & Manley, 2014). The compensatory advantage of HSI is thus the possibility of parallel on-line scanning of samples on a single object basis, which is impossible with other NIR technologies. However, the cost of HSI instrumentation and its added complexity should be also taken into consideration for practical applications.

Armstrong (2014) recently evaluated the potential of NIR spectroscopy to predict single-kernel protein content, reporting a value of RMSEP of 0.7%. Other papers dealing with protein prediction in single wheat kernels reported RMSECV values of 0.93–1.52% (Bramble et al., 2006), and RMSEP values from 0.46 to 0.72% (Delwiche, 1998) to 0.83% (Delwiche, 1995). These authors, however, used traditional NIR instruments and not a hyperspectral imager, where there is also an interference of the sample presentation, e.g. moving tray, possible shadows and light position effects. Thus, whereas we applied a different technique which implies a worse sample presentation, the prediction ability of our model was comparable to previous predictions. Our results are also in accordance with Bramble et al. (2006) for the observed apparent departure from the ideal slope at high protein content, which should be considered for samples with very high protein content.

Possible sources of error in protein prediction by NIR spectrometry can be attributed to the uncorrectable spectral non-linearities caused by kernel size and shape, analytical error for the reference measurement and the different wheat varieties used (Delwiche, 1998). Different wheat genotypes also imply differences in the relative abundance of amino acids and therefore differences in the nitrogen-to-protein ratio. Delwiche (1998) highlighted that the most important spectral region for protein measurement is between 1100 and 1400 nm, and that the accuracy of the model was lower when higher spectral regions were investigated. However, in our case the performance of the model improved when using more spectral bands.

In our case, we applied a HSI system with a push-broom method that can have advantages in terms of sample scanning, as individual measurements for a high number of wheat kernels can be acquired on-line.

**Fig. 4:**
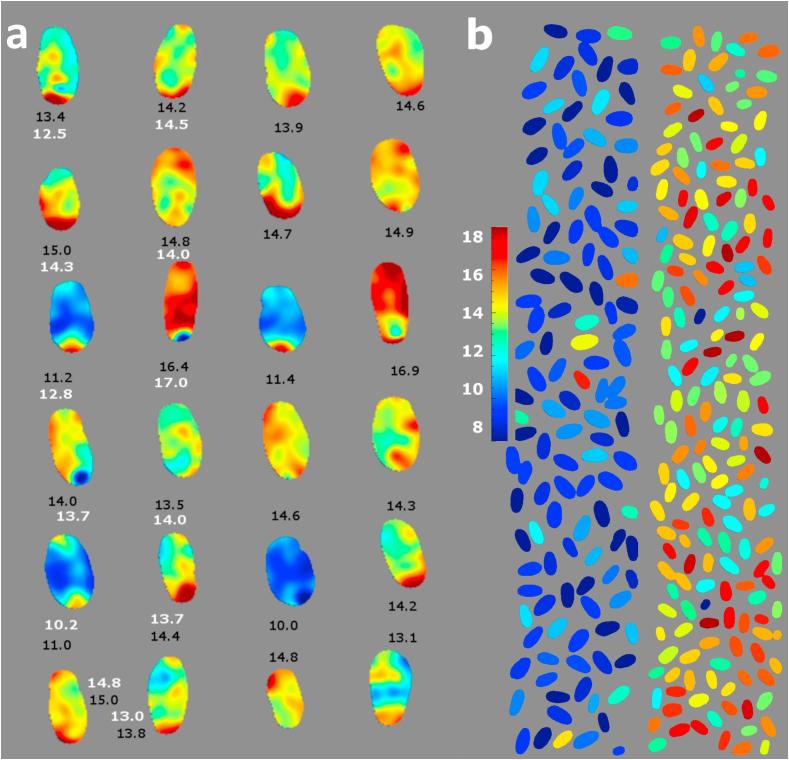
Application of the PLS regression protein calibration. **a**) Application at single pixel level, showing the dorsal (left; average: 12.7%) and ventral (right; average: 13.5%) sides of the same kernels. Reference protein measurement for the batch: 13.1%. **b**) Application by averaging the spectra for each kernel; Left: low protein sample, Viscount, reference protein content = 10.2%; predicted value = 9.7%. Right: high protein batch, DNS, reference protein content = 15.1%, predicted value = 14.6%. Black text; predicted values; white text: reference values (% “as-is”). For the colour identification please refer to the online colour version.

A potential benefit of the HSI approach is the ability to study protein variation within wheat kernels. Fig. 4a gives an example of the application of the best PLS model to a hyperspectral image on a single pixel basis, showing the predicted distribution of protein content for one of the sample batches (cultivar JB Diego, hard winter UK wheat). The predicted protein values are non-uniform within each grain, visualised by applying the calibration on each pixel of the hypercube. In particular, a consistently higher protein is predicted for the ends of the grain, particularly those where the germ is located. The effect was consistent for images of the same kernels taken in multiple orientations (not shown), and implies that this is a genuine non-uniformity, potentially in protein content, within the grains and not an artefact of the illumination or imaging conditions.

Through the application of the calibration on a HSI machine, it is possible to rapidly scan a considerable number of samples and predict the protein content on the average spectra from each kernel, as exemplified in Fig. 4b for two wheat samples.

We have verified this aspect, by applying the best protein calibration for four independent wheat batches, and obtaining hypercubes of over 100 kernels per batch, for which bulk protein measurements were then made by the Dumas method. The average predicted protein for the batches was within 0.1–0.8% of the reference measurement of the batch.

### 3.3 Effect of kernel presentation, hardness class and spectral region

As our dataset included a wide range of both hard and soft wheat varieties, it was of interest to study whether the sample presentation and hardness class influenced the PLS regression model for protein prediction. Moreover, we assessed the effect of longer wavelengths by excluding the higher spectral region to build a new model in the region 1000–1700 nm only, which is of interest for lower cost detectors. Table 3 shows the performances of the PLS models obtained by considering the effect of sample orientation, wheat classes and lower spectral region.

**Table 3:** Performances of the PLS models for protein prediction in single wheat kernels, by discriminating depending on (**a**) the orientation, (**b**) the hardness class and (**c**) for a restricted spectral range.

	Pre-processing	Variable	Calibration				Validation				PC	RPD	Samples	
			Slope	Bias	RMSEC	R^2^	Slope	Bias	RMSEP	R^2^			Cal.	Val.
**a)**	Raw data	Crease-up	0.774	0.008	0.990	0.774	0.743	0.029	1.048	0.728	15	1.97	1647	522
	SNV + 1st derivative		**0.824**	**0.000**	**0.868**	**0.824**	**0.803**	**0.024**	**0.958**	**0.773**	**13**	**2.15**		
	2nd derivative		0.757	0.026	1.028	0.757	0.775	0.010	1.091	0.705	10	1.89		
	Raw data	Crease-down	0.841	0.000	0.796	0.841	0.792	−0.062	0.958	0.768	18	2.15	1490	473
	SNV + 1st derivative		**0.834**	**0.000**	**0.817**	**0.834**	**0.804**	−**0.080**	**0.947**	**0.773**	**9**	**2.18**		
	2nd derivative		0.794	−0.002	0.885	0.794	0.751	−0.045	1.082	0.704	9	1.90		

**b)**	Raw data	Soft wheat	0.773	−0.004	0.703	0.773	0.711	−0.070	0.838	0.733	14	2.46	708	223
	SNV + 1st derivative		**0.812**	−**0.025**	**0.611**	**0.819**	**0.721**	−**0.059**	**0.790**	**0.764**	**11**	**2.61**		
	2nd derivative		0.790	−0.001	0.670	0.790	0.705	−0.052	0.841	0.731	11	2.45		
	Raw data	Hard wheat	0.791	0.022	0.923	0.791	0.766	0.107	0.996	0.763	17	2.07	1626	511
	SNV + 1st derivative		**0.824**	**0.000**	**0.852**	**0.824**	**0.826**	**0.032**	**0.901**	**0.806**	**12**	**2.29**		
	2nd derivative		0.744	−0.009	1.026	0.074	0.769	0.003	1.114	0.704	10	1.85		

**c)**	Raw data	<1700 nm	0.675	−0.009	1.192	0.675	0.668	0.013	1.185	0.648	14	1.74	3138	992
	SNV		**0.732**	**0.012**	**1.063**	**0.732**	**0.711**	**0.007**	**1.129**	**0.681**	**14**	**1.83**		
	MSC		0.747	0.013	1.034	0.747	0.728	−0.545	1.233	0.620	14	1.67		
	SNV + 1st derivative		0.688	0.000	1.159	0.688	0.702	−0.018	1.173	0.655	10	1.76		
	2nd derivative		0.703	−0.153	1.128	0.703	0.721	−0.008	1.187	0.647	10	1.74		

Number of spectral bands selected: 240 for a) and b); 107 for c). Abbreviations: RMSEC: Root mean square error of calibration; RMSEP: root mean square error of prediction; PC: principal component; MSC: multiplicative scatter correction; SNV: standard normal variate. Both first and second derivatives were calculated using Savitzky-Golay smoothing. The models shown in bold indicate the best performance among the groups. RMSEC and RMSEP are expressed in % (“as is” basis).

As reported in Table3a, the kernel position did not significantly influence the models’ performance. A slightly lower prediction error was observed when the kernels were placed crease-down, i.e. the dorsal side was analysed. However, the R^2^ values were similar and sample orientation had little effect on calibration performance and thus no improvement is obtained when building separate PLS models for the kernel position, which indicates that a unique calibration can be applied to wheat kernels with no need to place them in the same orientation. A similar comparison (Table3b) was seen for soft and hard wheat classes. In both cases, the best prediction model was obtained by applying SNV + 1st derivative treatment. The RMSE was 0.61% for the soft wheat class and 0.85% for hard wheat, for the calibration dataset, and the R^2^ values were similar for both calibration and validation datasets. However, as the performance is not much better for separate calibrations, there is little benefit in using them and a general model with applications to several wheat classes would be advantageous. In general, the grain processing industry would prefer a comprehensive PLS model that includes both hard and soft wheat classes, instead of having two separate calibrations.

The performance was also tested for a model using only the 1000–1700 nm wavelength region, to simulate detectors operating in a shorter spectral range and compare the improvement obtained in protein prediction when using HgCdTe detectors. Detectors such as InGaAs operating in this band are available at lower cost than those such as HgCdTe operating at longer wavelengths. Fox and Manley (2014) reported that it is interesting especially for protein to use sensors which are capable of detecting higher wavelengths, up to 2500 nm.

As shown in Table3c, the PLS regression built on NIR data with limited spectral range resulted in generally lower R^2^ and higher RMSE values. The RMSEC ranged from 1.03 to 1.19%, while RMSEP values were 1.13–1.23%. The best model was achieved with SNV treatment. Although the R^2^ for the restricted spectral region was reduced to 0.73 for the calibration and 0.68 for validation, this performance could still be useful at practical level for the rapid screening of kernel protein content, using a lower cost detector working in the spectral region 1000–1700 nm.

**Fig. 5:**
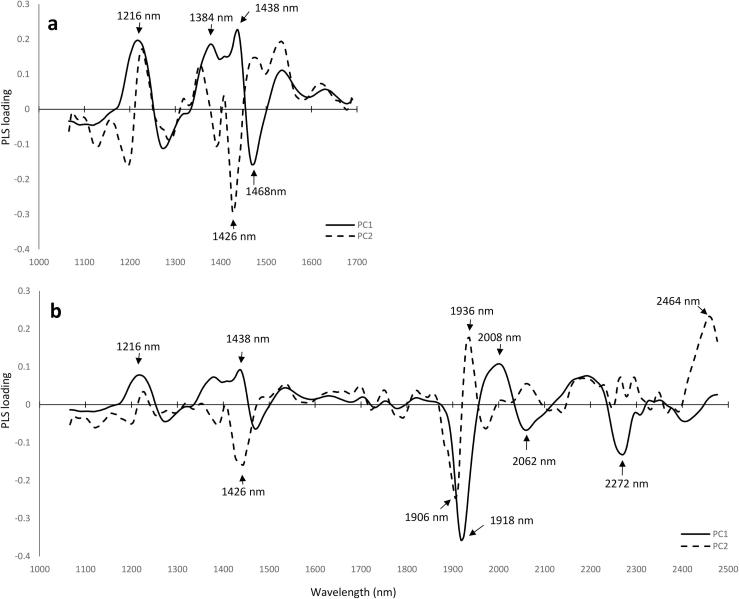
Loading weights of the first two factors for PLS protein prediction in the region 1000–1700 (**a**) and 1000–2500 nm (**b**), for the PLS models applying SNV + 1st derivative pre-processing calculation.

A comparison has been made of the PLS loading weights for the models using the full spectral region and the reduced one (Fig. 5). The main wavelengths contributing to PC1 are 1216, 1384, 1438 and 1468 nm. When the full spectrum is taken, a marked influence of the following wavelengths is also obtained: 1918, 2008, 2062 and 2272 nm. The most prominent feature was found at 1918 nm, which is attributed to a C=O second overtone, —CONH structure, and therefore indicating protein (Osborne, Fearn, & Hindle, 1993). In the case of PC2, the main influencing wavelengths were those at 1426, 1906, 1936 and 2464 nm. The 1936 nm wavelength has one of the highest scores, and this region represents the O—H combination of stretch and deformation, which is attributed to moisture. It is known that the C—H combination bands are mainly shown in the regions 1000–1100 nm and 2000–2500 nm, while the N—H combination bands absorb predominantly around 2000 nm (Osborne et al., 1993). For this reason, and also considering the fact that the HSI detector used gives more noisy spectra in the region 970–1060 nm, the most interesting region was above 1700 nm. In the overtone region, the N—H bond absorbs in the spectral range around 1500 nm. In the combination region, the N—H bond is mainly detected slightly below 2100 nm and around 2250 nm, and thus the main peaks at 2062 and 2272 were attributed to N—H vibration. The other peaks were mostly attributed to C—H combination bands (Shenk, Workman, & Westerhaus, 2001).

### 3.4 NIR prediction of kernel weight

Previous research reported on the possibility of predicting kernel weight based on NIR spectroscopy measurements, but for different types of grains, particularly maize and soybean (Agelet et al., 2012, Baye et al., 2006), and little information has been found in the literature on wheat kernel weight prediction by NIR or HSI and therefore no direct comparison can be made One paper has been found for wheat, where single kernel weight was predicted by NIR, with a reported RMSE of 2.8–4.0 mg, using a NIR instrument designed for single seed characterisation so that the light scattering was minimised (Armstrong, 2014).

The possibility of predicting kernel weight by HSI was tested for our dataset, which includes the average spectra for each kernel, and their reference weight measurements. Because the data were taken with an imaging system, measurements of kernel area were also available. As expected, the measured weight of single kernels correlated quite well with the area analysed by HSI, showing a Pearson correlation coefficient of 0.88, and RMSEC corresponding to an area of 2.70 mm^2^, for a mean area of 33.79 ± 5.54 mm^2^.

It was also noticed that the kernel area strongly depended on the kernel orientation, thus reducing the quality of the correlation with kernel mass. This effect would be particularly true when trying to continuously scan moving kernels, therefore the application of a calibration based on the spectral information may be of interest.

Table2b reports the performances of the PLS regression models for weight prediction using the NIR spectra, for several pre-processing methods. The R^2^ values of the model ranged from 0.62 to 0.68 for the calibration dataset, and 0.57 to 0.62 for the validation set. The best pre-processing technique was the baseline correction followed by the de-trending calculation, which gave a PLS model with the lowest calibration error of 6.8 mg and a validation error of 7.4 mg, for a range of kernel weights of 15.5–87.7 mg. The R^2^ value is acceptable at least for discrimination purposes into wheat kernel size classes.

Agelet et al. (2012) reported that working at shorter wavelengths, i.e. 900–1650 nm, did not give significantly different results in terms of prediction performance. In this spectral region, it has been suggested that the light scattering effect is increased, and this is correlated with soybean kernel size. Previous literature dealing with NIR prediction of small grain weight reported slightly better performances for soybean kernel weight prediction, i.e. R^2^ = 0.77–0.91 depending on the spectral pre-treatment, wavelength region selected and instrumentation used (Agelet et al., 2012).

Baye et al. (2006) predicted several compositional properties in maize kernels by NIR reflectance and transmittance spectroscopy. Interestingly, the authors reported that transmittance data cannot be used to predict kernel weight, while a good prediction capacity was found for the reflectance data However, there is an alternative route to estimate kernel weight when applying HSI, i.e. using the image analysis and therefore correlating the area of the kernel expressed as number of pixels with the weight. Both approaches might be valid and their application depends on the specific aim and needs at practical level.

## 4 Conclusions

We reported on a novel technology able to predict chemical properties of foods in a fast and non-destructive way also showing their spatial distribution, for the prediction of total protein content in whole wheat on a single kernel basis. We found a wide spread of protein content for over 2000 kernels analysed individually, from approximately 6–20%, with a standard deviation in protein content above 1.5% (range 0.5–3%) between kernels in each wheat batch. We successfully applied HSI for the visualisation of protein content in single wheat kernels, obtaining a statistical model with R^2^ above 0.8 and a prediction error below 1%. The model included samples sourced from a variety of genotypes and locations, and our calibration was effectively applied on both hard and soft wheats, including differences in the kernel orientation.

The spectral range of 1000–2500 nm was used for the general model, which gave a noteworthy improvement over the prediction achievable using shorter spectral range (1000–1700 nm), simulating InGaAs sensors. The prediction error for single kernel protein is greater than that typically achieved for the protein content of bulk samples by traditional NIR instruments, but is comparable with previous studies for single kernels. This provides additional information on sample uniformity, and the HSI approach provides the potential for rapid analysis of large numbers of kernels. The calibration error would be suitable for segregation of wheat kernels. Interestingly, a correlation was also obtained by HSI for the prediction of wheat kernel weight, although a better prediction from HSI was provided by kernel area.

While the single kernel approach is likely not to substitute bulk analysis for protein determination in wheat trading, it can bring several advantages for researchers, as it allows a faster and non-destructive method for single kernel protein analysis. The measurement of protein by HSI also has potential application to wheat breeding to select kernels according to their protein content.
